# Now you see me, now you don’t: verifying the absence of alien invasive yellow crazy ant *Anoplolepis gracilipes* in South Africa

**DOI:** 10.3389/finsc.2023.1176810

**Published:** 2023-06-07

**Authors:** Abusisiwe Ndaba, Thinandavha Caswell Munyai, Nokuthula Mbanyana, Simon van Noort, Charlene Janion-Scheepers

**Affiliations:** ^1^ Department of Biological Sciences, University of Cape Town, Cape Town, South Africa; ^2^ School of Life Sciences, University of KwaZulu-Natal, Pietermaritzburg, South Africa; ^3^ Research and Exhibitions Department, Iziko Museums of South Africa, Cape Town, South Africa

**Keywords:** *Anoplolepis gracilipes*, biosecurity, early detection, introduction pathway, invasive species, South Africa

## Abstract

*Anoplolepis gracilipes* is an invasive species that is a major threat to native ecosystems worldwide. It has been listed as one of the top 100 worst invasive species in the world and is well known for its negative impact on native arthropods and some vertebrates. This study aimed to confirm the presence or absence of *A. gracilipes* in some major South African harbours. We did so by surveying four harbours in the Western Cape and KwaZulu-Natal provinces, using pitfall trapping, yellow pan traps, and baiting. In addition, ant collections from Iziko Museums of South Africa (Cape Town, South Africa), University of KwaZulu-Natal (Pietermaritzburg campus, South Africa), Iimbovane Outreach Project (Stellenbosch University, South Africa), and AfriBugs CC (Pretoria, South Africa) were examined for specimens of *A. gracilipes*. The invasive species *A. gracilipes* was not detected from any of the sampled harbours during this study, nor in the main ant collections in South Africa. The only, and potentially erroneous published record of *A. gracilipes* in South Africa, is from Durban harbour and subsequent possibly erroneous citizen science observations are from other coastal sites such as Gansbaai, Knysna, Table Bay, and Kalk Bay. This is a positive outcome for conservation authorities as this species is highly invasive and, if introduced, will likely outcompete native fauna and result in ecosystem collapse. Although *A. gracilipes* was not detected in the samples from this study, early detection and eradication of this species should be prioritised. This can be achieved through existing pest monitoring programs at harbours, and continued border biosecurity measures.

## 1 Introduction

*Anoplolepis gracilipes* (1) is a well-known, widely distributed invasive species and has spread globally through human-mediated pathways (2). It has been recorded across the pacific tropics, from India to China, Japan, Australia, Chile, Mexico and California. Although the origin of this species is unknown (3), ecological niche modelling to reconstruct the ancestral distribution range suggests the origin of *A. gracilipes* might have been South Asia (4). Based on its current known distribution, *A. gracilipes* prefers warm and humid areas (3–5). It is known to thrive in highly disturbed habitats and areas with intermediate human activity. However, this species also inhabits undisturbed areas such as natural forests (2). The main introduction pathway of *A. gracilipes* is at ports and harbours as stowaways in containers and from transporting bulk materials (6, 7).

*Anoplolepis gracilipes* has been listed as one of the 100 worst invasive alien species in the world by the International Union for Conservation of Nature (IUCN) through its Invasive Species Specialist Group (ISSG) and Global Invasive species database (7). In South Africa, it is listed under NEMBA category 2b and South African Biodiversity Act, 2004 (Act No.10 of 2004). Species in this category must be controlled as no risk assessment has been done, nor is the distribution of the species known (8). Previous studies have investigated the spatial distribution patterns and population structure of *A. gracilipes* (4). This species has a high density of ground foraging workers and is numerically and behaviourally dominant (2). Its dominance facilitates its success in out-competing native ant species (9). The most severe ecological consequences of *A. gracilipes* include the displacement of native ants and other species of vertebrates and invertebrates (4, 9). This species also alters natural ecosystems’ structure, composition, and function (2). For example, on Christmas Island, *A. gracilipes* rapidly eliminated keystone species such as the red land crab (10), which caused major irreversible ecosystem disruption and made way for secondary invasions (11). The red land crab plays an important role in Christmas Island’s Forest ecosystem by facilitating litter breakdown and influencing forest composition by eating leaves and seedlings of rainforest trees (7). Regions predicted to be highly susceptible to *A. gracilipes* invasion include Asia, Australia, Africa, and South America (4). These areas should particularly focus on preventing the introduction of this invasive species.

Various records of *A. gracilipes* have been reported in South Africa (12, 13; https://www.inaturalist.org/observations/1160269; https://antmaps.org/?mode=species&species=Anoplolepis.gracilipes). The first published record of *A. gracilipes* was from Durban harbour (12). Other observations for this species are from Gansbaai, Knysna, Table Bay and Kalk Bay (**Supplementary Figure 1**) (13; https://www.inaturalist.org/observations/1160269; https://antmaps.org/?mode=species&species=Anoplolepis.gracilipes). Slingsby (13) suggested that this species has expanded its historical distribution into new areas of the Western Cape, which is alarming given the negative impact this species has on other species worldwide. However, no pictures or specimens are available for these records. This study aimed to verify the existing distributional records for the invasive species, *A. gracilipes* in South Africa and monitor its main introduction pathway (harbours). This was achieved by not only sampling ants at different harbours in South Africa but focusing on areas where this species was previously recorded and also examining ant museum collections in South Africa.

## 2 Methods and materials

### 2.1 Study area

Harbours are usually the first detection site of *A. gracilipes* in other regions (14). This study was conducted at four different harbours in South Africa, namely: Kalk Bay (34.1293° S, 18.4493° E) and V&A Waterfront (33.9050° S, 18.4204° E) in the Western Cape Province, and Durban (29.8723° S, 31.0249° E) and Richards Bay harbours (28.8000° S, 32.0833° E) in KwaZulu-Natal Province. Sampling sites were chosen based on previously recorded sites (Durban: 12; Gansbaai, Knysna, Table Bay, and Kalk Bay: 13; Kalk Bay: https://www.inaturalist.org/observations/1160269). The other records of *A. gracilipes* included Gansbaai and Knysna (15), but due to logistical constraints, these sites could not be sampled. Nevertheless, the four harbours sampled were representative of the major harbours and most confirmed records of this species in South Africa to date.

**Figure 1:**
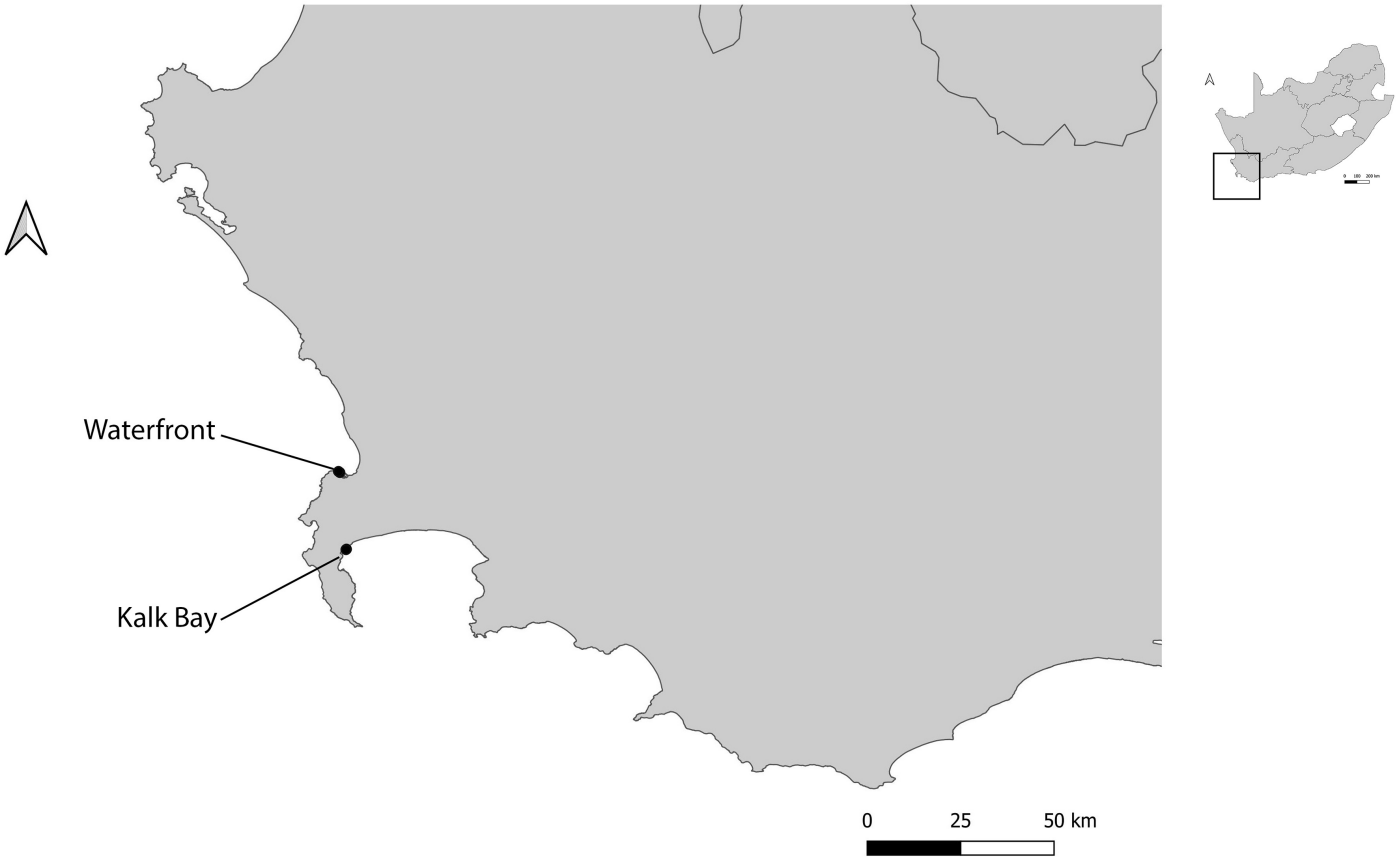
Location of different study sites used for ant sampling in two harbours (Kalk Bay and V&A Waterfront), Western Cape, South Africa.

**Figure 2:**
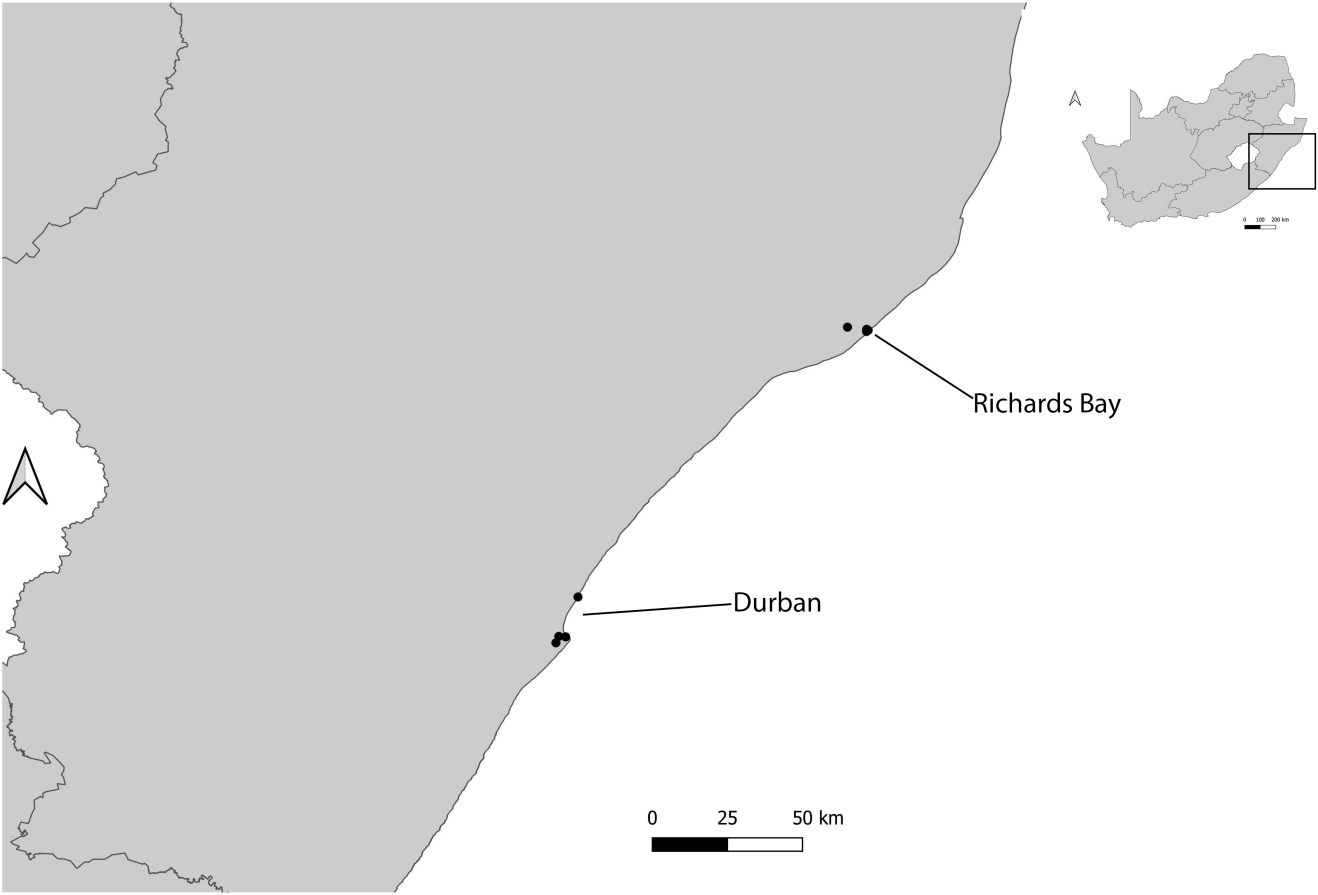
Location of different study sites used for ant sampling in two harbours (Durban and Richards Bay), KwaZulu-Natal, South Africa.

At each harbour, at least two sites surrounding the harbour were sampled. Where possible, these sites were replicated and sites with vegetation were selected as most ants need soil for nesting (16). These included: two sites in Kalk Bay (inside harbour and outside harbour); two sites in V&A Waterfront (Transnet building and helicopter pad) (**Figure 1**); five sites in Durban (Bayhead heritage site, Island and Channel View Park, South beach, Royal Natal view park and Umhlanga rocks) and four sites in Richards Bay (Port of Richards Bay, Pelican Island, Palm beach and Alkantstrand beach) (**Figure 2**). The study sites were characterized by different habitat types, such as grasslands, mangroves, sand dunes, and rocky shores (17). These site types were chosen as these spanned the range of habitat types available across the harbours (17).

### 2.2 Ant sampling and species identification

A combination of collection techniques was employed to collect all ant species present at each harbour. Ants were sampled using standardized pitfall trapping (18–20), yellow pan traps, and baiting. Where possible, sampling was done every two weeks for three months between June and September 2021. At each site, 10 pitfall traps were laid out in a sample grid (2 x 5) with 10 m spacing between traps. This resulted in 310 samples (**Supplementary Table 2**). Pitfall traps were half-filled with 100% propylene glycol that neither repels nor attracts insects (21) and were left open in the field for three days and three nights. In areas with concrete where pitfall traps could not be used, yellow pan traps were used with the same layout method as pitfall traps as an additional method to cover the whole area of the harbours (**Supplementary Table 2**). Yellow pan traps have also been used in other studies to sample ants (22–24).

In addition to these trapping methods, the baiting method was used across all study sites after the pitfall traps and yellow pan traps had been taken out. At the same sites as the pitfall traps and yellow pan traps, two card papers, one with tuna and one with peanut butter mixed with jam (used as attractants for ants), were laid out across all sites for one hour. These baits were found to be effective in attracting ants (25). All ants found at bait traps were collected with an aspirator and placed in vials containing 96% ethanol. Samples were processed and identified to genus in the laboratory using available keys (26) and AntWeb (www.antweb.org). Specimens were stored in 96% ethanol and voucher species were mounted. Where possible, species were confirmed using the Iziko Museums of South Africa’s reference ant collection. Those that could not be identified to species level were assigned as morphospecies. The second and third authors of the current study (ant experts) also confirmed the identifications. All specimens were labelled, catalogued and entered into the Iziko Museum of South Africa’s database (Specify 6 V6.7.01). The ant collection at Iziko Museums of South Africa (Cape Town, South Africa), University of KwaZulu-Natal (Pietermaritzburg campus, South Africa), Iimbovane Outreach Project (Stellenbosch University, South Africa), and AfriBugs CC (Pretoria, South Africa) were examined for specimens of *A. gracilipes.* This species is recognised by its monomorphic and remarkably long and slender yellow-brownish body of 4-5mm with a dark abdomen (2, 27), and its extremely long legs and antennae, with scapes longer than the body (27, 28).

### 2.3 Data analysis

Species accumulation curves were made using the specaccum() function in the vegan package in R V4.1.2 (29) to ensure sampling was done at an adequate level to capture resident biodiversity within a defined margin of error.

## 3 Results

In total, 10,041 specimens were sampled, comprising 66 species from 27 genera and five subfamilies (**Supplementary Table 1**). Myrmicinae was the most diverse and abundant subfamily with 10 genera, 45 species and 77% of the total abundance, followed by Formicinae with eight genera, 12 species and 18% of the total abundance. Dorylinae was the least diverse with two genera, two species and 0.5% of the total abundance. The most specious genera were *Tetramorium* (18 species), *Pheidole* (eight species) and *Monomorium* (five species). Genus *Pheidole* was most abundant in KwaZulu-Natal harbours, while *Tetramorium* was the most species-rich genus. The most abundant species in the Western Cape harbours were *Lepisiota capensis* and *Linepithema humile*, while *Tetramorium* was the most species-rich genus. The invasive species *A. gracilipes* was not detected from any of the sampled harbours during this study, nor in the main ant collections in South Africa. However, in the Western Cape harbours, we collected a lot of *Linepithema humile* which is a major problem in the Western Cape province and none in the KwaZulu-Natal harbours (**Supplementary Table 1**).

The four accumulation curves from various harbours show that the increase in sampling sites resulted in an increased number of species (**Supplementary Figure 2**). Most of the accumulation curves reached or nearly reached a horizontal asymptote indicating sufficient sampling, although the Richards Bay curve indicated that more sampling might be needed at this site in the future.

## 4 Discussion

In this study, four harbours were sampled in the Western Cape and KwaZulu-Natal provinces in South Africa, including the exact locations where *A. gracilipes* was previously recorded. However, no specimens of this species were found. Although the species could have been accidentally introduced to South Africa at ports and harbours, no specimens, images, or drawings exist of the records of *A. gracilipes* from South Africa. Therefore, the specimens determined as *A. gracilipes* could have been misidentified. Although other species of *Anoplolepis* were found in harbours and could have been misidentified as *A. gracilipes*, *A. gracilipes* (Formicinae) is morphologically more similar to the minor workers of *Camponotus maculatus* (Formicinae) and to some species of *Leptomyrmex* (Dolichoderinae) than to other *Anoplolepis* species. These ant genera have long limbs and a similar-sized, slender body. However, *A. gracilipes* and *C. maculatus* can be distinguished from *Leptomyrmex* by the presence of an acidopore, which is often easily overlooked. The photo of *A. gracilipes* on iNaturalist is presented by a drawing and not the actual specimen collected (**Supplementary Figure 1**). A character like an acidopore is not clearly visible without magnification, thus the identification remains questionable. *Anoplolepis gracilipes* and *C. maculatus* can be separated based on the number of antennal segments. In *A. gracilipes*, antennae has 11 segments, whereas in *C. maculatus* has 12 segments. These two species can also be separated based on the shape of the mesosoma.

The characters that separate *A. gracilipes* from other species in the genus *Anoplolepis* include their monomorphic (26) and remarkably long and slender yellow-brownish body of 4-5mm with a dark abdomen (2). The legs and antennae are extremely long, with scapes longer than the body (27, 28). In Kalk Bay harbour, where *A. gracilipes* was recorded in 2012 (P. Slingsby, https://www.inaturalist.org/observations/1160269; **Supplementary Figure 1**), the alien invasive *Linepithema humile* (Argentine ant) was found to be the dominant ant species across all Western Cape province harbours in this study, occurring in almost all pitfall traps (**Supplementary Table 1**).

Since the detection of *A. gracilipes* at Kalk Bay in 2012, the site was transformed into a parking area (P. Slingsby, https://www.inaturalist.org/observations/1160269; **Supplementary Figure 3**), and this species was not found again. This could have been a result of *A. gracilipes* not being able to compete with the abundant *L. humile* (Argentine ants) present at this site (13). *Linepithema humile* is one of the most widespread ant invasive species in South Africa that has successfully invaded at least six of the nine provinces in South Africa (15, 30). This species is also listed as one of the world’s 100 worst invaders (7, 31). It is well known for its aggressiveness and displacement of native invertebrates and small vertebrates (2, 32, 33). *Anoplolepis gracilipes* has eliminated the red land crab, a keystone species, in parts of the island resulting in significant ecosystem disruption in Christmas Island (11). On other islands such as Seychelles, when *Anoplolepis gracilipes* occurred in high abundance, it took over the nests, preventing scooty terns from nesting, thus leading to the death of chicks (11). Several native ant species have been successfully displaced by *L. humile*, disrupting plant-ant mutualism (34, 35). Thus, if there was a small population of *A. gracilipes* present at this site, it could have potentially been displaced by *L. humile*. Moreover, the latest study by Lee and Scotty Yang (2) suggested that the South African population of this species has been eradicated.

Once alien species are established, their management is costly and is considerably more than the prevention of new invasive species (36). The measures taken to prevent losses or enable restoration of ecosystem services in an invaded area can be very costly. For example, an estimated US$300 billion per year is spent as a result of invasive species in the United States, British Isles, Australia, South Africa, India, and Brazil alone (8). In the vineyards in Western Cape, chemical stem barriers were effective in most ant pests (37). However, chemical stem barriers are ineffective in controlling species of the genus *Anoplolepis* (38). Although most chemicals are registered for use in controlling invasive species, the negative impact on other organisms caused by the use of these chemicals far exceeds their cause of action (39). Therefore, the early detection of invasive species is critical to increasing the chances of successful management (36). This is especially prudent given the impact *A. gracilipes* had on the wine and grape industry in countries in Central America (40). The Western Cape wine industry plays a huge role in the country’s economy, with a contribution of about R31 billion to the gross domestic product and more than 160 000 employment opportunities, which is 57% and 62% contribution to the country’s total wine industry contribution, respectively (https://www.wosa.co.za/The-Industry/Statistics/World-Statistics/). This is because the wine industry of South Africa is more concentrated in the Western Cape (https://www.wosa.co.za/The-Industry/Statistics/World-Statistics/). However, ants such as *L. humile* and some species of *Anoplolepis* are a major problem in vineyards of the Western Cape province (38).

Ants, in particular *A. gracilipes*, feed on honeydew produced by aphids and other scale insects, thereby protecting them from infestation promoted by a build-up of honeydew and protecting aphids from predators (41). Through the consumption of honeydew by ants, the survival of honeydew-producing pests is increased (42). This increases the damaging effects on crops through pest outbreaks in agroecosystem (42). Furthermore, in the Cape Floristic Region, most fynbos plants are dispersed by ants (31). Therefore, the presence of *A. gracilipes* may also negatively impact fynbos seed dispersal. Despite the absence of this species in this study, prevention of the introduction of *A. gracilipes* in South Africa should be prioritized. The economic and ecological impacts of this species can be reduced through quarantine programs in susceptible areas (3).

Ants are sensitive to changing climatic conditions such as temperature, water stress, and wind (43). However, invasive species are known to have broader tolerances to warming and drying conditions than indigenous species, as found for other soil-dwelling invertebrates (44). The foraging activity of *A. gracilipes* is largely affected by ambient temperature, with the highest activity levels at 26°C and 30°C (45). This species prefers moist tropical lowlands. However, there is still potential for possible invasion in arid regions, mainly because this species can still thrive in urban and irrigated areas (3). This is therefore why ongoing monitoring should be done throughout southern Africa. This can easily be accomplished through existing pest monitoring programs at harbours, and through standard border control monitoring. Monitoring greenhouses and plant nurseries is recommended as alien ants can be introduced in greenhouses, for example through soil movement in potted plants (46).

## Conclusions

Although *A. gracilipes* was not detected in the samples from this study, ongoing monitoring is essential to ensure the early detection and eradication of this species. Additional taxonomic information should be provided to persons at ports of entry. Other global monitoring programs should include mesic tropical, subtropical, and warm temperate mainland islands that are most susceptible to being invaded by *A. gracilipes* (47). In addition, the use of citizen science platforms like *iNaturalist* is a useful research tool for the early detection of severe pests such as *A. gracilipes*. However, it is recommended that records from sources such as iNaturalist and antmaps (https://antmaps.org/; https://www.inaturalist.org/) need to be carefully verified before being included in species lists. For future studies, more sampling should be done along provincial borders, including the Eastern Cape borders and neighbouring countries to South Africa (Namibia and Mozambique), which were not sampled in this study.

## Data availability statement

The original contributions presented in the study are included in the article/**Supplementary Material**. Further inquiries can be directed to the corresponding author.

## Author contributions

All authors contributed to the study’s conception and design. Field sampling was performed by AN, TM, and CJ-S. Laboratory analyses and statistical analysis were performed by AN. The first draft of the manuscript was written by AN, and all authors commented on previous versions. All authors read and approved the final manuscript.
