# Supplementary material for: Now you see me, now you don’t: verifying the absence of alien invasive yellow crazy ant Anoplolepis gracilipes in South Africa
Source: Front Insect Sci. 2023 Jun 7;3:1176810. doi: 10.3389/finsc.2023.1176810 (PMC10926371; doi:10.3389/finsc.2023.1176810)
Supplement: Supplementary file 1 [file DataSheet_1.docx]

# Supplementary Figures and Tables

## Supplementary Tables

**Supplementary Table 1**: Mean ( ± SE) species richness across harbours in Western Cape and KwaZulu-Natal harbours, South Africa.

|  |  | | | |
| --- | --- | --- | --- | --- |
| **Species names** | **Durban harbour** | **Kalk Bay** | **Richards Bay** | **Waterfront harbour** |
| *Anochetus* sp.1 | 0 | 0 | 0.143 ± 0.331 | 0 |
| *Anoplolepis custodiens* | 81.941 ± 327.765 | 0 | 0 | 0 |
| *Camponotus maculatus* | 0.118 ± 0.471 | 0 | 0.143 ± 0.331 | 0 |
| *Camponotus* sp.1 | 1.118 ± 1.529 | 0.5 ± 0.5 | 0.857 ± 1.059 | 0 |
| *Camponotus* sp.2 | 0 | 0 | 0.286 ± 0.661 | 0 |
| *Cardiocondyla* sp.1 | 0.765 ± 2.184 | 0 | 1.571 ± 2.118 | 0.125 ± 0.331 |
| *Cardiocondyla* sp.2 | 0.059 ± 0.235 | 0 | 0 | 0 |
| *Cardiocondyla* sp.3 | 0.059 ± 0.235 | 0 | 0 | 0 |
| *Crematogaster liengmei* | 0 | 0 | 0.143 ± 0.331 | 0 |
| *Crematogaster rectinota* | 0.294 ± 0.956 | 0 | 0 | 0 |
| *Crematogaster* sp.1 | 0.118 ± 0.471 | 0 | 0 | 0 |
| *Diplomorium* sp.01 | 1 ± 2.870 | 0 | 0 | 0 |
| *Dorylus helvolus* | 0 | 0 | 5.571 ± 12.898 | 0 |
| *Hypoponera* sp.1 | 0 | 0 | 0.286 ± 0.661 | 0 |
| *Lepisiota* sp.1 | 0.529 ± 1.144 | 0 | 47 ± 108.807 | 10.125 ± 15.186 |
| *Lepisiota spinosior* | 0.118 ± 0.471 | 0 | 0 | 0 |
| *Leptogenys* sp.1 | 0 | 0 | 0.143 ± 0.331 | 0 |
| *Linepithema* humile | 0 | 25.5 ± 7.5 | 0 | 3 ± 4.330 |
| *Mesoponera* sp.1 | 0.588 ± 1.751 | 0 | 0.571 ± 1.323 | 0 |
| *Messor* sp.1 | 0 | 0 | 0 | 0.375 ± 0.484 |
| *Monomorium junodi* | 0.059 ± 0.235 | 0 | 8.857 ± 18.342 | 0 |
| *Monomorium* sp.1 | 1.471 ± 4.704 | 0 | 4.286 ± 7.172 | 1 ± 2.645 |
| *Monomorium* sp.2 | 16.765 ± 23.598 | 0.5 ± 0.5 | 7.429 ± 12.578 | 2.875 ± 4.512 |
| *Monomorium* sp.3 | 3.353 ± 7.522 | 35 ± 17 | 0 | 0.375 ± 0.696 |
| *Monomorium* sp.4 | 0.765 ± 2.101 | 0 | 0 | 4.875 ± 5.134 |
| *Myrmicaria natalensis* | 5.588 ± 17.915 | 0 | 4.571 ± 9.500 | 0 |
| *Nylanderia natalensis* | 0.176 ± 0.706 | 0 | 0 | 0 |
| *Nylanderia* sp.1 | 1.529 ± 3.550 | 0 | 0 | 0 |
| *Paratrechina* sp.1 | 1.706 ± 3.339 | 0 | 0 | 0 |
| *Pheidole crassinoda* | 0 | 0 | 0.143 ± 0.331 | 0 |
| *Pheidole* sp.1 | 6.118 ± 13.625 | 0 | 0 | 0 |
| *Pheidole* sp.2 | 0.588 ± 2.353 | 0 | 0.143 ± 0.331 | 0 |
| *Pheidole* sp.3 | 0.353 ± 1.412 | 0 | 5 ± 11.571 | 3 ± 3.464 |
| *Pheidole* sp.4 | 0.588 ± 2.353 | 0 | 6.143 ± 14.221 | 0 |
| *Pheidole* sp.5 | 1.941 ± 5.525 | 0 | 0 | 0 |
| *Pheidole* sp.6 (*megacephala* gp.) | 258.294 ± 604.415 | 0 | 226.714 ± 250.081 | 0.625 ± 0.992 |
| *Pheidole* sp.7 (*megacephala* gp.) | 0.059 ± 0.235 | 0 | 0 | 0 |
| *Plagiolepis* sp.1 | 0.529 ± 1.882 | 0 | 0 | 0 |
| *Polyrhachis* sp.1 | 0.235 ± 0.941 | 0 | 0 | 0 |
| *Solenopsis* sp.1 | 0.353 ± 0.681 | 0 | 0.143 ± 0.331 | 0 |
| *Solenopsis* sp.2 | 0.412 ± 1.647 | 0 | 0 | 0.125 ± 0.331 |
| *Strumigenys* sp.1 | 0.059 ± 0.235 | 0 | 0 | 0 |
| *Tapinolepis* sp.1 | 0 | 0 | 0.571 ± 1.321 | 0 |
| *Tapinoma* sp.1 | 0.353 ± 1.185 | 0 | 1 ± 1.965 | 0 |
| *Technomyrmex pallipes* | 11 ± 19.369 | 0 | 0.143 ± 0.331 | 0 |
| *Tetramorium gabonense* | 5.706 ± 10.374 | 0 | 10.714 ± 17.449 | 0 |
| *Tetramorium notiale* | 0.353 ± 0.836 | 0 | 0 | 0 |
| *Tetramorium sericiventre* | 2.765 ± 5.504 | 0 | 6.857 ± 7.794 | 6.125 ± 6.918 |
| *Tetramorium setigerum* | 1.235 ± 4.453 | 1.5 ± 1.5 | 2.429 ± 4.854 | 0.375 ± 0.484 |
| *Tetramorium setuliferum* | 0 | 0 | 0.143 ± 0.331 | 0 |
| *Tetramorium simillimum* | 2.176 ± 7.031 | 0 | 1.714 ± 3.606 | 0 |
| *Tetramorium* sp.1 | 0.294 ± 1.176 | 0 | 1.857 ± 2.913 | 0 |
| *Tetramorium* sp.10 | 0 | 0 | 0.286 ± 0.661 | 0 |
| *Tetramorium* sp.11 | 0 | 0 | 0 | 2.25 ± 4.465 |
| *Tetramorium* sp.12 | 0 | 0 | 2.714 ± 5.243 | 0 |
| *Tetramorium* sp.13 | 0.706 ± 2.371 | 0 | 0 | 0.5 ± 1.323 |
| *Tetramorium* sp.2 | 0 | 0 | 0.286 ± 0.661 | 0 |
| *Tetramorium* sp.3 | 0 | 0 | 0.143 ± 0.331 | 0 |
| *Tetramorium* sp.4 (*squaminode* group) | 0.176 ± 0.706 | 0 | 0 | 0 |
| *Tetramorium* sp.5 (*squaminode* group) | 1.235 ± 4.941 | 0 | 0 | 0 |
| *Tetramorium* sp.6 (*squaminode* group) | 1.235 ± 4.466 | 0 | 0 | 0 |
| *Tetramorium* sp.7 | 0.059 ± 0.235 | 0 | 0 | 0.125 ± 0.331 |
| *Tetramorium* sp.8 | 6.118 ± 24.471 | 0 | 0 | 0 |
| *Tetramorium* sp.9 | 0.059 ± 0.235 | 0 | 0 | 0 |

**Supplementary Table 2: Table showing the number of sites and replicates of each harbour and coordinates.**

| **Habour** | **Sites** | **Replicate** | **Coordinates** |
| --- | --- | --- | --- |
| Kalk Bay | Inside harbour  Outside harbour | 1  1 | 34.129743S 18.449165E  34.129743S 18.449165E |
| Waterfront  Durban  Richards Bay | Transnet building  Helicopter pad  Bayhead  South Beach  Umhlanga Rocks  Royal Natal Yacht Club  Island Channel View Park  Alkantstrand  Palm Beach  Pelican Island  Port of Bay | 1  1  1  2  3  4  1  2  1  2  1  2  1  1  1  1  2  3  1 | 33.903948S 18.429956E  33.900755S 18.425862E  30.489211S 31.027767E  30.489736S 31.025917E  30.151339S 31.021267E  30.477681S 31.021225E  30.442986S 31.076667E  30.449258S 31.082492E  30.219517S 31.156306E  30.220656S 31.156589E  30.443947S 31.037583E  30.447731S 31.044831E  30.494036S 31.060028E  29.341753S 32.155942E  29.341822S 32.170864E  29.332222S 32.156150E  29.336267S 32.147278E  29.332322S 32.152611E  29.322061S 32.049103E |

## Supplementary Figures


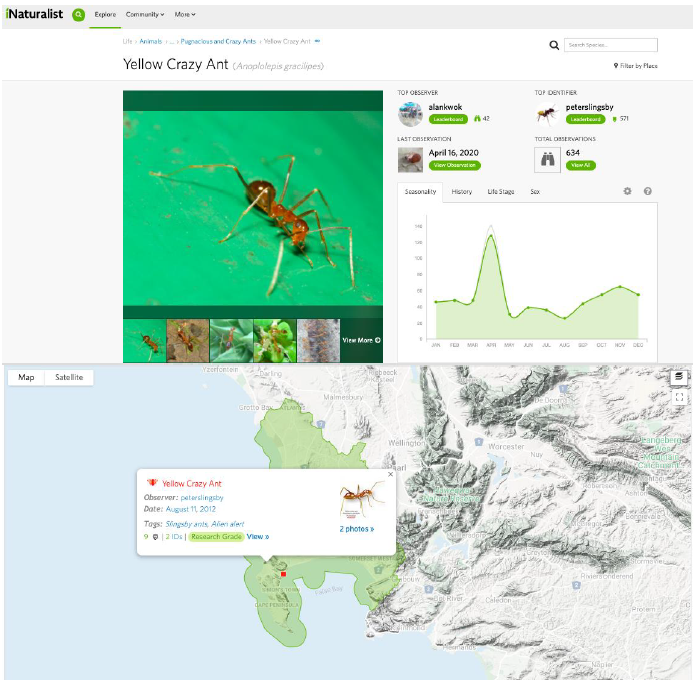


**Supplementary Figure 1:** Record from <https://www.inaturalist.org/observations/1160269>, accessed 10 January 2022.


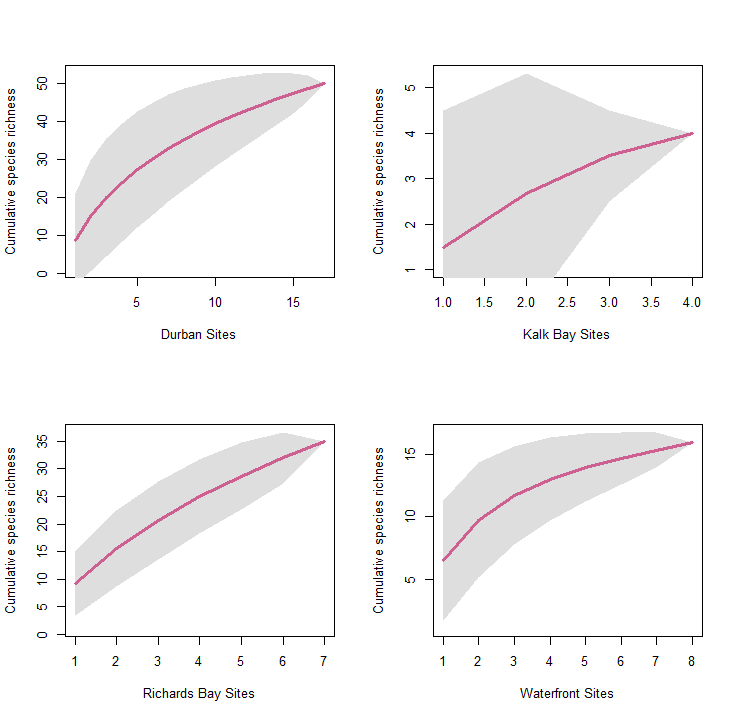


**Supplementary Figure 2:** Cumulative species richness of sampling in various sites of the four harbours (Durban, Richards Bay, Kalk Bay, and V&A Waterfront).


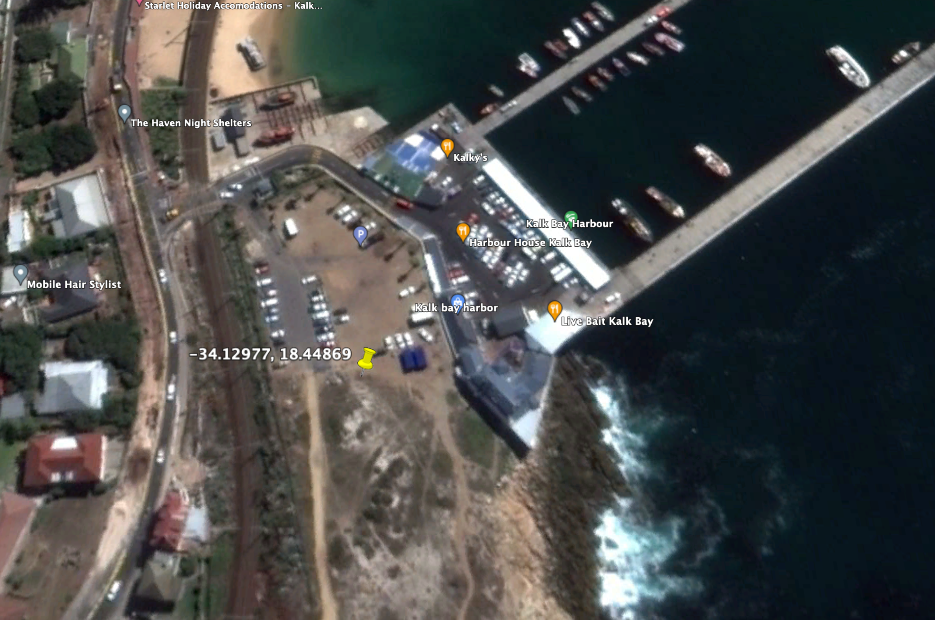


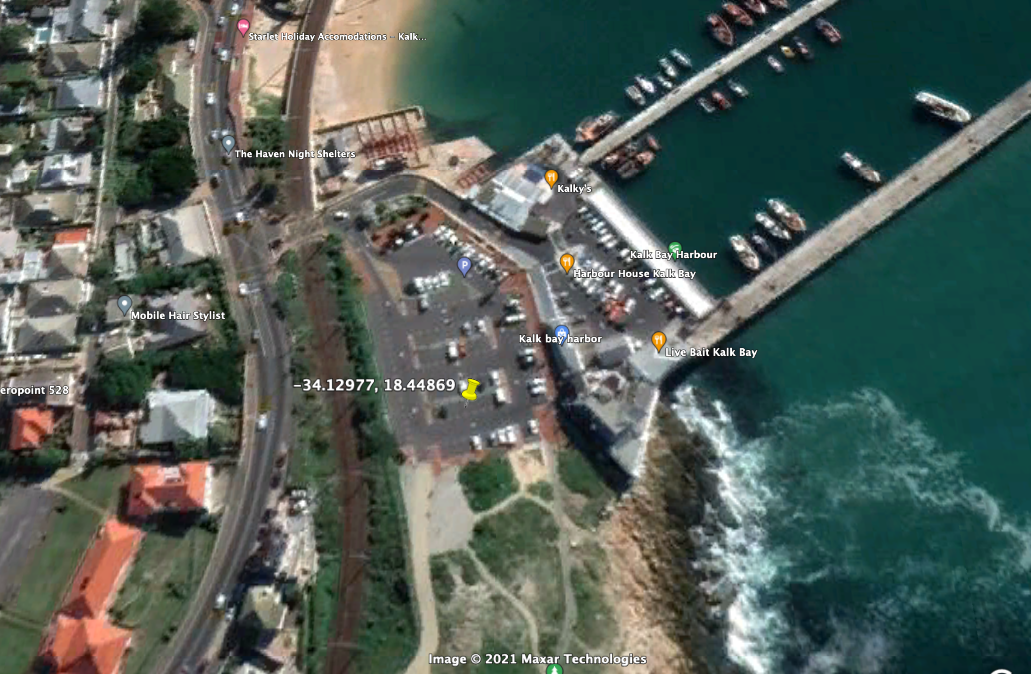


**Supplementary Figure 3:** Site of the record of *A. gracilipes* in Kalk Bay site in 2013 (top) and 2021 (bottom) showing the parking area construction.
